# Effect of Substrate Support on Dynamic Graphene/Metal Electrical Contacts

**DOI:** 10.3390/mi9040169

**Published:** 2018-04-07

**Authors:** Jihyung Lee, Xiaoli Hu, Andrey A. Voevodin, Ashlie Martini, Diana Berman

**Affiliations:** 1 Materials Science and Engineering, University of North Texas, Denton, TX 76203, USA; JiHyungLee@my.unt.edu (J.L.); Andrey.voevodin@unt.edu (A.A.V.); 2 Mechanical Engineering, University of California Merced, Merced, CA 95343, USA; xhu@ucmerced.edu; 3 Advanced Materials and Manufacturing Processes Institute, University of North Texas, Denton, TX 76203, USA

**Keywords:** graphene, electrical conductivity, contact evolution, atomic force microscopy

## Abstract

Recent advances in graphene and other two-dimensional (2D) material synthesis and characterization have led to their use in emerging technologies, including flexible electronics. However, a major challenge is electrical contact stability, especially under mechanical straining or dynamic loading, which can be important for 2D material use in microelectromechanical systems. In this letter, we investigate the stability of dynamic electrical contacts at a graphene/metal interface using atomic force microscopy (AFM), under static conditions with variable normal loads and under sliding conditions with variable speeds. Our results demonstrate that contact resistance depends on the nature of the graphene support, specifically whether the graphene is free-standing or supported by a substrate, as well as on the contact load and sliding velocity. The results of the dynamic AFM experiments are corroborated by simulations, which show that the presence of a stiff substrate, increased load, and reduced sliding velocity lead to a more stable low-resistance contact.

## 1. Introduction

The exceptional electrical, thermal, chemical, mechanical, and optical properties of two-dimensional (2D) materials are continuing to find new applications in many different fields [1,2,3,4]. One of the main future directions for 2D materials is flexible electronics [5], where controllable electrical performance [6] is complemented by high mechanical strength [7], wear resistance [8], and corrosion inhibition [9]. Graphene, a one-atom thick carbon material, is one of the primary 2D materials being considered for future device fabrication [10,11]. However, complex designs of functional structures often require incorporating several materials [12], and creating and maintaining a stable contact between these materials is challenging.

Multiple efforts have been focused on understanding the effect of contact geometries and material properties on electrical contact at nano- and micro-scale junctions [13]. Significant work has also been performed to understand the effect of normal force on the contact resistance of such junctions [14,15,16,17]. However, there are still challenges related to applying this understanding to device design [18,19,20]. To address these challenges, solutions have been proposed to improve permanent contacts between graphene and electrode materials by e-beam nano-welding [21], or incorporating adhesion layers or changing the contact geometries [22]. However, in the case of dynamic or periodically actuated electrical contacts, which are essential for devices such as microelectromechanical systems (MEMS), controlling electrical current flow in the contact zone is not a straightforward procedure, even for well-studied noble metal interfaces [23,24,25].

Conductive atomic force microscopy (AFM) is one of the traditional techniques for characterizing the electrical properties of surfaces [26]. For 2D materials, this approach has been implemented for testing the effectiveness of the oxygen reduction procedure in reduced graphene oxide films [27], monitoring the tunneling current through few monolayer thick hexagonal boron nitride films [28], and studying the surface potential and electron transport mechanisms in molybdenum disulfide nanoflakes [29]. Conductivity mapping of chemically and mechanically exfoliated graphene films using AFM indicated higher electrical conductivity of the graphene films in the absence of structural or chemical defects [30]. It was also shown that, when strained to about 6%, graphene may exhibit a step function variation in contact with copper; the effect was attributed to the graphene’s work function changing to match the work function of copper [31]. Electron mobility of graphene can also be affected by the substrate; free-standing graphene or graphene transferred onto inert surfaces demonstrates higher electron mobility than graphene deposited on SiO_2_ surfaces, due to less interference of confined charges by the adjusted media [32].

Here, using conductive AFM complemented by atomistic simulations, we explore how the electrical dynamic contact between graphene and a noble metal is affected by the contact load, substrate material and the sliding velocity. The major aspect distinguishing this work from previously reported graphene/metal contact studies [33,34] is our focus on the dynamic behavior of non-stable, periodically actuated contacts.

## 2. Materials and Methods

**Figure 1:**
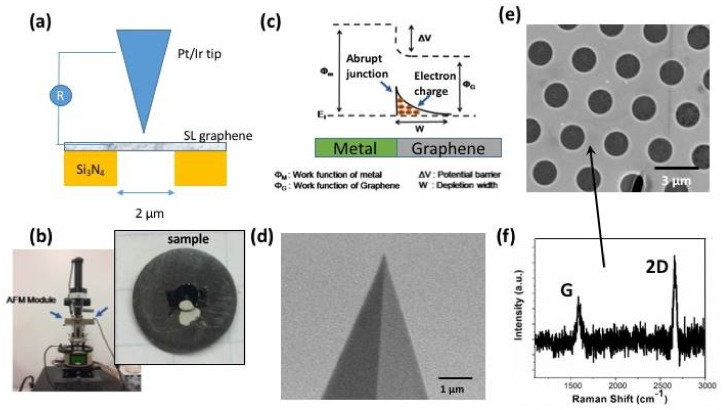
Summary of the experimental setup. (**a**) Schematic of the AFM measurements of electrical contact evolution for Pt/Ir tip and graphene performed for free-standing and supported graphene and (**b**) photograph of the sample assembly. (**c**) Metal–graphene band diagram for the contact. (**d**) SEM image of the conductive AFM tip. (**e**) SEM image of the single layer graphene transferred on a silicon nitride substrate with holes. (**f**) Raman analysis confirming single layer graphene presence after the graphene transfer.

To characterize changes in electrical contact, AFM measurements (Figure 1a,b) were performed for graphene coming into contact with a conductive metal-coated tip. Note, that, from the graphene–metal band diagram (Figure 1c), graphene is an independent electronic sheet that shows non-classical band bending interactions with metallic contact. This creates an abrupt transition and potential barrier for any charge carrier tunneling. The carriers generate large contact resistance because of charge build up at the band edge [35]. The measurements were performed using Pt/Ir conductive tip (purchased from Bruker, Billerica, MA, USA) with ~20 nm in diameter (Figure 1d). First, single layer graphene films were transferred onto a nonconductive silicon nitride substrate with 2 µm diameter holes; see Figure 1e. The silicon nitride substrate was chosen to eliminate any possible cross conductivity contribution from the substrate. The graphene, which was chemical vapor deposition (CVD) grown on a copper foil, was covered with a spin-coated 200 nm thick polymethyl(methacrylate) (PMMA) film. Then, the copper was etched in a copper etchant and the resulting graphene with the PMMA film on top was transferred onto the silicon nitride grid (Ted Pella, Redding, CA, USA, silicon nitride grid with holes). The PMMA was removed with a warm acetone bath. Complete removal of the PMMA layer and the single-layer nature of the graphene film covering the holes in the substrate were confirmed using scanning electron microscopy (SEM) and Raman spectroscopy with 534 nm green laser [36], as shown in Figure 1f. Specifically, SEM images indicate that graphene transfer resulted in uniform coverage of most of the holes, and the relative intensity of the Raman 2D peak (at ~2700 cm^−1^) and G peak (at ~1560 cm^−1^) confirm the single-layer nature of the film.

The samples were attached to the insulating quartz substrate using a ceramic paste, and the grounding connection was made on the edge of the graphene-on-Si_3_N_4_ sample using a conductive silver paint. This sample geometry was created to allow the electrical current to travel from the metal tip to the graphene, and then laterally across the graphene film to the contacting pads. The electrical resistance of the contact pads and silver paint used for grounding was confirmed to be less than 1 ohm, which is much less than the >500 ohms measured from the AFM experiments. Therefore, the contribution of resistance due to grounding could be eliminated from the measurements, and the effect of the dynamically actuated contact between the AFM tip and the graphene sample monitored directly.

AFM measurements were performed in ambient atmosphere conditions (relative humidity 30%) using a Bruker Multimode AFM in contact mode with a conductive platinum/iridium tip (spring constant of 0.1 N/m). Specifically, two types of measurements were performed. First, the evolution of the electrical contact between the Pt/Ir tip and free-standing and supported graphene was tested in static mode while increasing the applied load. The maximum load was limited to 13.5 nN, which remained well below critical loads required for free-standing CVD grown graphene rapture (order of 2000 nN) or for inelastic deformation under AFM nanoindentation [7,37]. The applied bias voltage varied from −2 V to 2 V. and the maximum electrical current flow was limited to a maximum of 1 µA to prevent local heating-induced failure of the tip and attachment of the graphene film. In the second type of measurement, the electrical current was monitored during scanning of the tip at a constant normal load on the free-standing and supported graphene areas while applying a constant bias voltage of 0.5 V.

## 3. Results and Discussion

**Figure 2:**
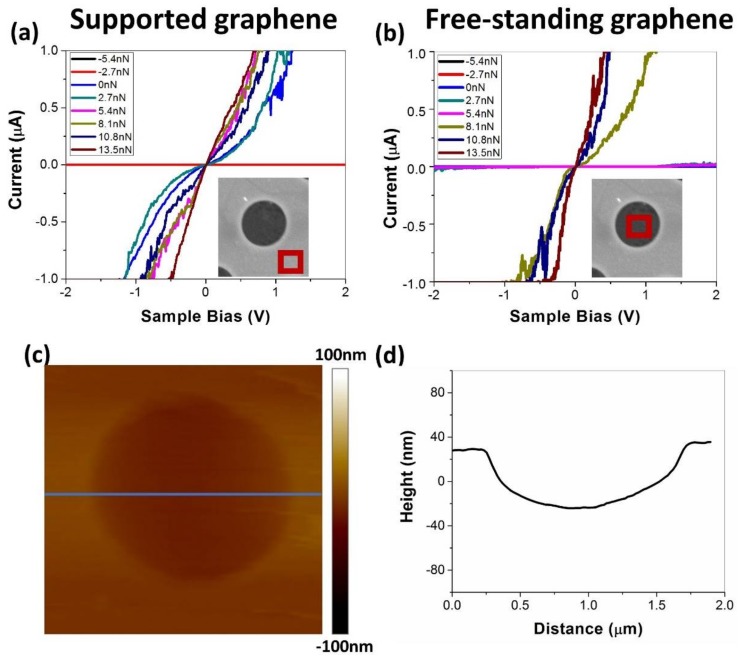
I–V characteristics of (**a**) supported graphene and (**b**) free-standing graphene as a function of the applied load. In the case of the supported graphene, larger current is observed at the lower loads. The 2.7 nN steps were selected to provide a uniform distribution of applied loads. (**c**) Height profile scan indicates (**d**) ~40 nm sagging of graphene in the free-standing area resulting from the transfer procedure.

Figure 2 summarizes the static electrical current measurements (I–V characteristics) for the supported and free-standing areas of graphene as the contact evolves only due to the influence of the changing contact load during data acquisition. Interestingly, at low applied load (up to 2.7 nN for the supported graphene and up to 8.1 nN for the free-standing graphene), the I–V characteristics for both free-standing and supported graphene demonstrate Schottky behavior. Previously, it was reported that the presence of oxygen bonded to carbon atoms directly, or in the form of hydroxide groups, introduces a finite-energy band gap in graphene structures [38,39]. Therefore, we attribute the observed nonlinear current vs applied voltage dependence to instability of the contact between graphene and tip, resulting in the presence of a small gap and leading to the nonlinear tunneling current effect. Additional contributions to nonlinearity may arise from the presence of OH groups on the graphene surface, due to the humid air environment in which all tests were performed [39]. Increasing the applied load results in a larger contact area [40], and thus, smaller contact resistance [13]. As the load is increased, the contamination molecules on the graphene surface can be pushed outside of the contact [41]; the contact becomes more stable and eventually transitions to linear ohmic behavior, traditionally observed for metal contacts [42]. Previously, nonlinear I–V characteristics of graphene–metal static contacts were related to metal doping of graphene [43,44]; however, in the experiments performed here, the metal contacts are periodic and of a short duration, which limits the doping effect. We note that comparable trends were observed for measurements with graphene on gold obtained with a different transfer technique (results not shown), which suggests the findings are independent of the substrate material and transfer process.

Although the supported and free-standing graphene exhibit some similar behaviors, there are differences between these two cases. Most significantly, a larger load is required to obtain substantial current on the free-standing graphene. For example, we observe significant current flow for the supported graphene at a load of 0 nN, while the free-standing graphene requires a load 8.1 nN to achieve this. We hypothesize that this difference is due to the stability of the contact, which is poorer for the free-standing graphene, due to the flexibility of the material.

**Figure 3:**
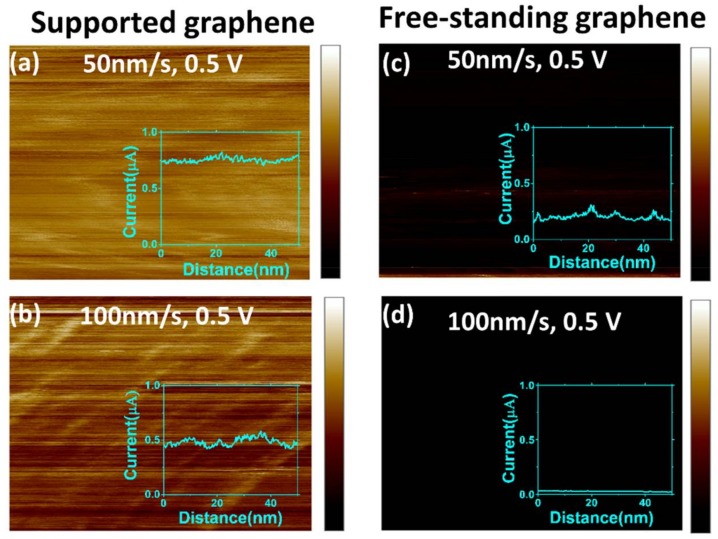
Conductive measurements during scanning of the graphene sample. Detailed 100 nm scans of the supported graphene area at scan speeds of (**a**) 50 nm/s and (**b**) 100 nm/s show that current was higher at the slower scan speed. In the case of free-standing graphene, overall conductivity is substantially lower both for (**c**) slow scanning and (**d**) faster scanning than for the supported graphene. In the case of the free-standing graphene, current is substantially reduced to ~0.2 μA at a scanning velocity of 50 nm/s and to 0.01–0.02 μA at 100 nm/s. The applied load was 5.4 nN for all results shown. The color scale bars cover the range from 0 up to 1.2 μA.

In order to further explore the stability of the metal/graphene contact, we performed dynamic electrical current mapping by scanning the conductive tip on the graphene surface, both on the supported and free-standing graphene areas. In the case of the free-standing graphene, the scanning area was selected to be close to the center of the membrane (100 nm × 100 nm). The load was fixed at 5.4 nN and the voltage bias at 0.5 V. Figure 3 shows the results of the observations during scanning.

The results demonstrate a similar trend of lower conductivity (lower electrical current is measured) on free-standing graphene than supported graphene (compare Figure 3a,c). Also, the observed current is lower during these dynamic measurements than for the stationary contact measurements at a similar applied load (Figure 2). This may be explained by instability of the contact during scanning. To further test the hypothesis that scanning affects the conductivity, we mapped the electrical current with two-times larger scanning velocity (Figure 3b,d). On both supported and free-standing graphene, the electrical contact between graphene and the tip was less stable at the faster scan speed, leading to reduced electrical current.

**Figure 4:**
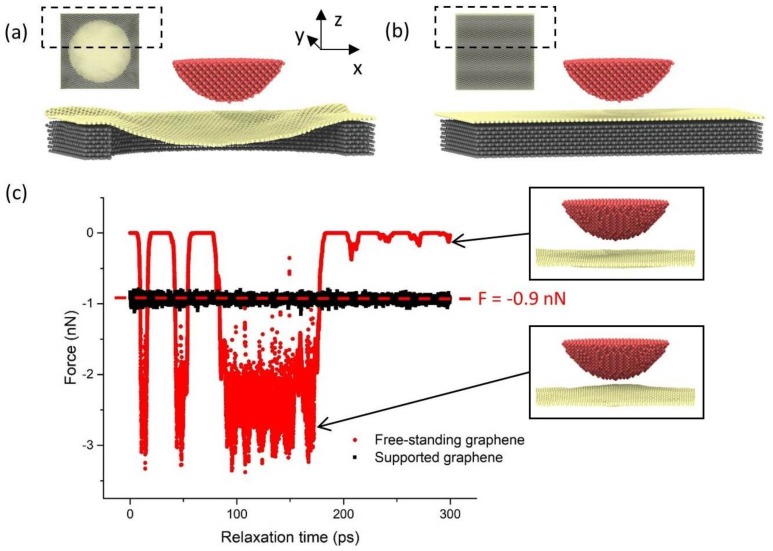
Snapshots of the models of (**a**) free-standing graphene and (**b**) supported graphene, where the main figures show perspective views of half of the model systems and the insets show bottom views. The regions identified by the dashed box in the insets correspond to those shown in the perspective views. (**c**) The interaction force between the tip and substrate as a function of relaxation time for free-standing graphene and supported graphene, where the dashed red line shows the average interaction force for both models. The insets show representative snapshots of the tip and free-standing graphene layer when they are in (bottom inset) and out of (top inset) contact.

To investigate our hypothesis that the difference between the contact conductance for free-standing and supported graphene is due to the contact stability, we developed atomistic models of these two systems. The models consisted of a diamond tip (radius 3 nm, height 2 nm) and a graphene layer that was either suspended by a gold substrate with 12 nm diameter hole (free-standing graphene membrane) or supported by a gold substrate without hole (supported graphene), shown in Figure 4a,b, respectively. In both models, the in-plane dimensions of the substrate were 15.2 nm × 14.6 nm. In the model of free-standing graphene, the sagging of the graphene observed in experiment (Figure 2d) was captured by introducing a graphene sheet that was 1% larger in the in-plane directions than the hole dimensions. In both models, the top five atomic layers of the tip were treated as a rigid body. The bottom two layers of the substrate and the atoms at the perimeter of the graphene layer were kept fixed throughout the simulation in order to constrain the movement of the system.

The embedded-atom method (EAM) potential was applied to simulate the Au–Au interactions [45] and the adaptive intermolecular reactive empirical bond order (AIREBO) potential was used to describe interactions within the tip and graphene layer [46]. For the Au–C interactions, the Morse potential was used with potential parameters D_0_ = 0.00832 eV, r_0_ = 0.387035 nm and α = 1.25707. The Lennard–Jones potential was used to model interactions between C atoms in the tip and the graphene layer, where the parameters were taken from [46].

The initial separation between the bottom of the tip apex and the fixed edge of the graphene layer in the z-direction was approximately 0.9 nm, a distance at which the interaction force between the tip and graphene layer was negligible. The model system was first equilibrated at 300 K for 20 ps. The temperature was controlled by applying Nosé–Hoover thermostat to all the non-constrained atoms. The tip was then moved at a speed of 10 m/s towards the graphene layer. At different vertical positions, the tip movement was stopped and the system was relaxed for 300 ps.

The interaction force between the tip and graphene as a function of relaxation time for the tip at a representative vertical position is shown in Figure 4c. For both the free standing and supported graphene cases, the average interaction force was −0.9 nN. However, it can be seen that, due to the instability of the contact for the free-standing graphene, there were large fluctuations in the instantaneous force. In addition, the force alternated between zero and non-zero, indicating intermittent contact between the tip and substrate (see insets to Figure 4c). This behavior was observed with the free-standing graphene model at other tip positions as well, but not with the supported graphene. This unstable contact could explain the experimental observation that there was lower conductivity for free-standing graphene than supported graphene at the same normal load (Figure 3a,c).

## 4. Conclusions

In conclusion, the effect of the substrate on the electrical performance of graphene/metal contact at a range of applied contact loads was investigated. Our results demonstrate more stable contact when graphene is supported by the substrate. When the support for the graphene is removed, as in case of free-standing graphene, the contact loses stability and contact resistance increases. This was observed in both static and dynamic contact measurements. For static contacts, the contact resistance for free-standing graphene at low contact loads was on average 5 times larger than that of supported graphene. For dynamic contacts, the free-standing graphene contact resistance was up to 4 times larger at slow 50 nm/s scanning, and more than 10 times larger at faster 100 nm/s scanning, when compared to the substrate-supported graphene. To test the hypothesis that the stability of the contact could explain the difference between the conductivity on free-standing and supported graphene, we developed atomistic models of these two systems. The simulations results indicated that the same normal load could be achieved with constant contact for the supported graphene and intermittent contact with the free-standing graphene. This behavior could correspond to lower conductivity at the same load, as observed in the experiment. Overall, our study emphasizes the important role of the contact stability in determining graphene/metal electrical conductance, where the substrate can have a pronounced effect on stability and therefore conductance of such contacts. This study also highlights the effects of contact mechanical loading and sliding velocity on current transport at graphene/metal contacts, which are critical for applications with dynamic or periodically actuated electrical contacts.
